# Cannabinoid 2 receptor attenuates inflammation during skin wound healing by inhibiting M1 macrophages rather than activating M2 macrophages

**DOI:** 10.1186/s12950-018-0201-z

**Published:** 2018-12-04

**Authors:** Yu Du, Peng Ren, Qi Wang, Shu-Kun Jiang, Miao Zhang, Jiao-Yong Li, Lin-Lin Wang, Da-Wei Guan

**Affiliations:** 1 Department of Forensic Pathology, China Medical University School of Forensic Medicine, No.77, Puhe Road, Shenyang North New Area, Shenyang, 110122 Liaoning Province People’s Republic of China; 2 Department of Forensic Medicine, Criminal Investigation Police University of China, Shenyang, 110854 China; 3 Collaborative Laboratory of Intelligentized Forensic Science, Shenyang, 110033 China; 4 Department of Forensic Pathology, School of Forensic Medicine, Southern Medical University, Guangzhou, 510515 China

**Keywords:** Cannabinoid 2 receptor, Macrophage polarisation, Inflammation, Skin wound healing

## Abstract

**Background:**

The anti-inflammatory properties of the cannabinoid 2 receptor (CB2R) in injury and inflammatory diseases have been widely substantiated. Specifically, the anti-inflammatory effect of CB2R may be achieved by regulating macrophage polarisation. Several research findings suggested that the activation of CB2R could attenuate inflammation by reducing pro-inflammatory M1 macrophage polarisation and promoting anti-inflammatory M2 polarisation. However, considering CB2R inhibits fibrosis and M2 promotes fibrosis, that the activation of CB2R may lead to an increase in M2 macrophages seems contradictory. Therefore, we hypothesised that the activation of CB2R to attenuate inflammation is not achieved by up-regulating M2 macrophages.

**Methods:**

We established an incised wound model using mouse skin and used this to evaluate the effect of CB2R agonists (JWH133 or GP1a) and an antagonist (AM630) on wound healing. At various post-injury intervals, we used western blot analysis, immunofluorescence staining, enzyme-linked immunosorbent assay and quantitative reverse transcription polymerase chain reaction assays to determine CB2R protein expression, M1/M2 macrophage infiltration, and the protein and gene expression of M1/M2-associated markers and cytokines in skin lesions.

**Results:**

Activation of CB2R significantly reduced M1 macrophage infiltration and slightly increased M2 macrophage infiltration. Similarly, gene expression and protein levels of M1-associated markers and cytokines (interleukin [IL]-6, IL-12, CD86 and inducible nitric oxide synthase) were significantly down-regulated after CB2R agonist administration; in contrast, markers and cytokines were increased in the CB2R antagonist–treated group. Conversely, the administration of agonists slightly increased gene expression and protein levels of M2-associated markers and cytokines (IL-4, IL-10, CD206 and arginase-1 [Arg-1]); however, a statistical significance at most time points post-injury was not noted.

**Conclusion:**

In summary, our findings suggested that during incised skin wound healing in mice, increased levels of CB2R may affect inflammation by regulating M1 rather than M2 macrophage subtype polarisation. These results offer a novel understanding of the molecular mechanisms involved in the inhibition of inflammation by CBR2 that may lead to new treatments for cutaneous inflammation.

## Background

Skin wound healing is a dynamically complex and multistage pathological process that is precisely regulated by multiple cells and cytokines with different physiological functions. Such a process involves three important stages: inflammation, fibrosis, and tissue remodeling [1, 2]. An inflammatory reaction is the first response after injury, and fluctuations in inflammation have a significant effect on wound healing. Exploring the regulatory mechanisms involved in inflammation will be conducive to discovering optimal methods for cutaneous wound therapy.

The endocannabinoid system (ECS), including endogenous ligands, cannabinoid receptors, and synthesising and degrading enzymes of endogenous ligands [3], has attracted a great deal of interest in terms of research on its multiple physiological and pharmacological functions. Cannabinoid receptors include cannabinoid 1 receptor (CB1R) and cannabinoid 2 receptor (CB2R) [4]. CB2R belongs to the G-protein–coupled receptor family, and is induced by active inflammation in both humans and mice [5]. It is mainly expressed in cells associated with innate immunity, such as microglia, astrocytes, neutrophils, macrophages, and myofibroblasts [6–8]. Studies in recent years have demonstrated that CB2R plays a key anti-inflammatory role in many inflammatory diseases and autoimmune diseases, such as inflammatory bowel disease [5], inflammatory kidney disease [9], periodontitis [10] and neuroinflammation [6, 11]. Our previous findings suggested that CB2R exhibited specific temporal changes in neutrophils, macrophages and myofibroblasts in a mouse model of an incised skin wound [7], and ameliorated wound healing by attenuating inflammation [8]. However, the mechanism by which CB2R reduces inflammation and promotes tissue repair in the course of skin wound healing is still not completely clear.

Macrophages, as key regulatory cells in the inflammatory response, play an important role in phagocytosing pathogens and necrotic tissue, and secreting a series of pivotal cytokines [12, 13]. Macrophages have a highly plastic phenotype depending on the immune microenvironment. The extremes of this phenotypic spectrum include classically activated macrophages (M1 phenotype, pro-inflammatory defensive role) and alternative activated macrophages (M2 phenotype, anti-inflammatory and tissue-repair roles) [14]. Accumulated evidence suggests that an imbalance of M1/M2 is associated with the process of inflammation in many diseases, such as chronic hyperinsulinemia [15], diabetic nephropathy [16] and radiation-induced lung fibrosis [17]. Therefore, regulating the polarisation of macrophages may be a new therapeutic method for overcoming inflammatory disease.

Several research groups have shown that the selective activation of CB2R can attenuate pro-inflammatory M1 macrophage polarisation and increase anti-inflammatory M2 polarisation [18–21]. However, considering CB2R inhibits fibrosis [8, 22, 23] while M2 promotes fibrosis [17, 24], the activation of CB2R to increase levels of M2 macrophages appears contradictory. Therefore, we hypothesised that the activation of CB2R to attenuate inflammation is not achieved by up-regulating M2 macrophage levels. Instead, we suggest that the activation of CB2R and the promotion of M2 polarisation may be different mechanisms of inhibiting inflammation.

To verify our hypothesis, a mouse incised skin wound model was developed and subsequently treated with CB2R agonists (JWH133 or GP1a) and an antagonist AM630. CB2R protein levels, M1/M2 macrophage infiltration, and the gene expression and protein level of M1/M2-associated markers and cytokines in skin lesions at different post-injury intervals were assessed. We found that CB2R attenuates inflammation during skin wound healing by inhibiting M1 macrophages rather than by activating M2 macrophages.

## Methods

### Animal groupings and drug treatments

A total of 105 healthy male 8-week-old BALB/c mice (Department of Laboratory Animal Science of China Medical University, Shenyang, China), each weighing 25 ± 3 g, were used in our experiments. All mice were housed individually in a temperature-controlled animal colony room with a 12 h light/dark cycle, and had access to commercial mouse chow and distilled water ad libitum.

An animal model of an incised cutaneous wound was developed based on our previous reports [7]. Briefly, all mice were anaesthetised using 2% sodium pentobarbital (intraperitoneal [i.p.] injection, 45 mg/kg). Subsequently, under sterile conditions, a 1.5-cm–long incision was made with a scalpel in the skin layer of the central dorsum of 100 mice. Five mice without an incised injury were used as a control (nonsurgical) group.

The post-injury treatment of mice was according to previously published methods [8, 22]. Briefly, mice that underwent surgery were randomly divided into four groups (vehicle group, JWH133 group, GP1a group, and AM630 group; 25 mice in each group) and administered an i.p. injection daily of either vehicle (5% DMSO/2% Tween-80/93% physiological saline), JWH133 (CB2R agonist dissolved in vehicle; Tocris Bioscience), GP1a (CB2R agonist dissolved in vehicle; Tocris Bioscience), or AM630 (CB2R antagonist dissolved in vehicle; Tocris Bioscience) at a dose of 3.0 mg/kg/day, respectively. Vehicle, JWH133, GP1a or AM630 were administered in parallel to the excisional operation and on each subsequent day until the day prior to sacrifice.

Mice were sacrificed at 1, 3, 5, 7 or 9 d post-injury (five mice for each time point in each group) after an overdose of 2% sodium pentobarbital (i.p. injection, 135 mg/kg). An area of 1.5 cm × 1.0 cm of skin centred on a wound was harvested from each mouse. The same area of skin was harvested from control mice. Half of each specimen was used for morphological analyses, and the other half for western blotting, quantitative reverse transcription (qRT)–PCR and enzyme-linked immunosorbent assay (ELISA).

### Protein preparation and western blots

Skin tissues were homogenised at 4 °C with a sonicator in RIPA buffer (KGP9100, KeyGEN Biotech Co., Ltd., Nanjing, China) containing protease and phosphatase inhibitors. The homogenates were centrifuged three times at 12,000×g for 30 min at 4 °C and liquid supernatants were collected for each sample. The protein concentration of each sample was detected using the bicinchoninic acid method. Aliquots of the supernatants were mixed in an equal volume of 6 × electrophoresis sample buffer, and boiled for 5 min at 90 °C. Protein samples (50 μg) were separated on a 12% sodium dodecyl sulfate–polyacrylamide electrophoresis gel and transferred onto polyvinylidene fluoride (PVDF) membranes (Millipore, Billerica, MA, USA). After being blocked with 5% fat-free milk at RT for 2 h, PVDF membranes were incubated, respectively, with a rabbit anti-CB2R polyclonal primary antibody (1:250 dilution; sc-25,494, Santa Cruz Biotechnology, CA, USA) and mouse anti–GAPDH monoclonal antibody (1:2500 dilution, sc-32,233; Santa Cruz Biotechnology, Inc) at 4 °C overnight. The following day, after being washed with Tris-buffered saline–Tween 20 for 2 h, membranes were incubated with horseradish peroxidase (HRP)-conjugated goat anti-rabbit antibody (1:5000 dilution; sc-2004; Santa Cruz Biotechnology) for 2 h at RT. Blots were visualised using western blotting luminol reagent (sc-2048; Santa Cruz Biotechnology) and a Electrophoresis Gel Imaging Analysis System (MF-ChemiBIS 3.2, DNR Bio-Imaging Systems, Jerusalem, Israel). Subsequently, band densities were semi-quantitatively analysed using Image J software (Image J 1.48 v, National Institutes of Health, Bethesda, MA, USA).

### Immunofluorescence staining

Immunofluorescence staining was performed to identify M1 or M2 phenotypes of macrophages. Briefly, deparaffinised sections were blocked with 5% bovine serum albumin (BSA) and incubated with rat anti-CD86 monoclonal antibody (1:200 dilution, 11–0862-82; Thermo Fisher Scientific, Waltham, MA, USA) or goat anti-CD206 polyclonal antibody (1:200 dilution, PA5–46994; Thermo Fisher), and rabbit anti-F4/80 monoclonal antibody (1:100 dilution, ab100790; Abcam, Cambridge, UK) overnight at 4 °C. Then, the sections were incubated with Alexa Fluor® 647 donkey anti-rat IgG (1:100 dilution, abab150155; Abcam) or Alexa Fluor® 488 donkey anti-goat IgG (1:100 dilution, abab150129; Abcam) and Alexa Fluor® 488 donkey anti-rabbit IgG (1:100 dilution, abab150073; Abcam) at RT for 2 h. After sufficient washing, nuclei were stained with DAPI (Sigma Chemical Company, St. Louis, MO, USA) for 10 min. Finally, stained sections were observed and photographed by fluorescence microscopy (DM4000 B; Leica Microsystems, Wetzlar, Germany). A positive signal was not detected when sections were incubated with normal rat non-immune IgG or phosphate buffered saline (PBS) as negative controls.

M1 (CD86-positive and F4/80-positive) or M2 macrophages (CD206-positive and F4/80-positive) were counted independently by two pathologists in five sections from each sample of skin lesion (five non-contiguous microscope fields for each section at 400-fold magnification) using a Leica DM400 B microscope.

### qRT–PCR assays

In order to further confirm the effects of CB2R on macrophage polarisation, the relative mRNA expression levels of M1/M2-associated markers and cytokines were measured by qRT–PCR.

The specific methods and procedures used have been described previously [22]. Briefly, total RNA was isolated from skin specimens with TRIzol (10296–028; Thermo Fisher Scientific) according to the manufacturer’s instructions. Optical density (OD) values of each RNA sample were measured by ultraviolet spectrophotometer. The RNA was reversely transcribed into cDNA using PrimeScript™ RT reagent Kit (RR037A; Takara Biotechnology, Shiga, Japan). The cDNA synthesis was performed in a 20-μL reaction mixture. The resulting cDNA was used for qRT–PCR with sequence-specific primer pairs for interleukin (IL)-4, IL-6, IL-10, IL-12, CD86, CD206, arginase-1 (Arg-1), inducible nitric oxide synthase (iNOS) and β-actin (Table 1). PCR amplification was performed by an Applied Biosystems 7500 Real-time PCR System (Thermo Fisher Scientific) using SYBR® PrimeScript™ RT-PCR Kit (RR081A; Takara Biotechnology). The qPCR thermal cycling was performed as follows: one cycle at 95 °C for 30 s, followed by 40 cycles of 95 °C for 5 s and 60 °C for 34 s, and one cycle of 95 °C for 15 s, 60 °C for 30 s and 95 °C for 15 s for fluorescence signal acquisition. The loading control used was β-actin. The Ct value was inversely proportional to the original template number. Relative quantification was performed using the comparative Ct (2^-ΔΔCt^) method, in which the ΔCt value represented the difference in Ct between the target genes in different groups and that of the internal reference gene, *β-actin*.

**Table 1:** Primer sequences of biomarkers detected in the present study

Biomarker	Primer		Product size (bp)	GenBank ID
*IL-4*	Forward	AACGTCCTCACAGCAACGAA	164	NM_021283
	Reverse	AGGCATCGAAAAGCCCGAAA		
*IL-6*	Forward	GTCCTTCCTACCCCAATTTCCA	151	NM_031168
	Reverse	CGCACTAGGTTTGCCGAGTA		
*IL-10*	Forward	TGGGTTGCCAAGCCTTATCG	118	NM_010548
	Reverse	TTCAGCTTCTCACCCAGGGA		
*IL-12A*	Forward	CTGCCGGCTATCCAGACAAT	168	NM_008351
	Reverse	TGGCCAAACTGAGGTGGTTT		
*CD86*	Forward	CTTACGGAAGCACCCACGAT	147	NM_019388
	Reverse	TGTAAATGGGCACGGCAGAT		
*CD206*	Forward	TTCCATCGAGACTGCTGCTG	168	NM_008625
	Reverse	CCAGAGGGATCGCCTGTTTT		
*iNOS*	Forward	CTATGGCCGCTTTGATGTGC	111	NM_010927
	Reverse	TTGGGATGCTCCATGGTCAC		
*Arg1*	Forward	ACATTGGCTTGCGAGACGTA	109	NM_007482
	Reverse	ATCACCTTGCCAATCCCCAG		
*β-actin*	Forward	ACCTTCTACAATGAGCTGCG	147	NM_007393
	Reverse	CTGGATGGCTACGTACATGG		

To eliminate any potential contamination, negative controls were also performed with double distilled water instead of cDNA during each run. No amplification product was detected. The qPCR procedure was repeated at least three times for each sample.

### ELISA assays

Tissue specimens were homogenised in 10 volumes of PBS, and then homogenates were centrifuged three times at 12,000×g for 30 min at 4 °C to remove tissue residue. The levels of IL-4, IL-6, IL-10, IL-12, CD86, CD206, iNOS and Arg-1 were measured in tissue extracts using commercially available ELISA kits (CSB-E04634m, CSB-E04639m, CSB-E04594m, CSB-E04600m, CSB-E08544m, CSB-EL014782MO, CSB-E08326m, CSB-EL002005MO; Cusabio, Wuhan, China) according to the manufacturer’s instructions. The results were averaged and expressed as picograms per milligram (pg/mg) of tissue. The ratios of IL-12/IL-10 and iNOS/Arg-1 protein levels were analysed.

### Statistical analysis

Data was expressed as the mean ± standard deviation (SD) and analysed using SPSS 13.0 for Windows. Two-way ANOVA with a Bonferroni post hoc test was used for data analysis. *P* < 0.05 was considered to indicate a statistically significant difference.

## Results

### Changes in CB2R protein expression level after agonist or antagonist administration

**Fig. 1:**
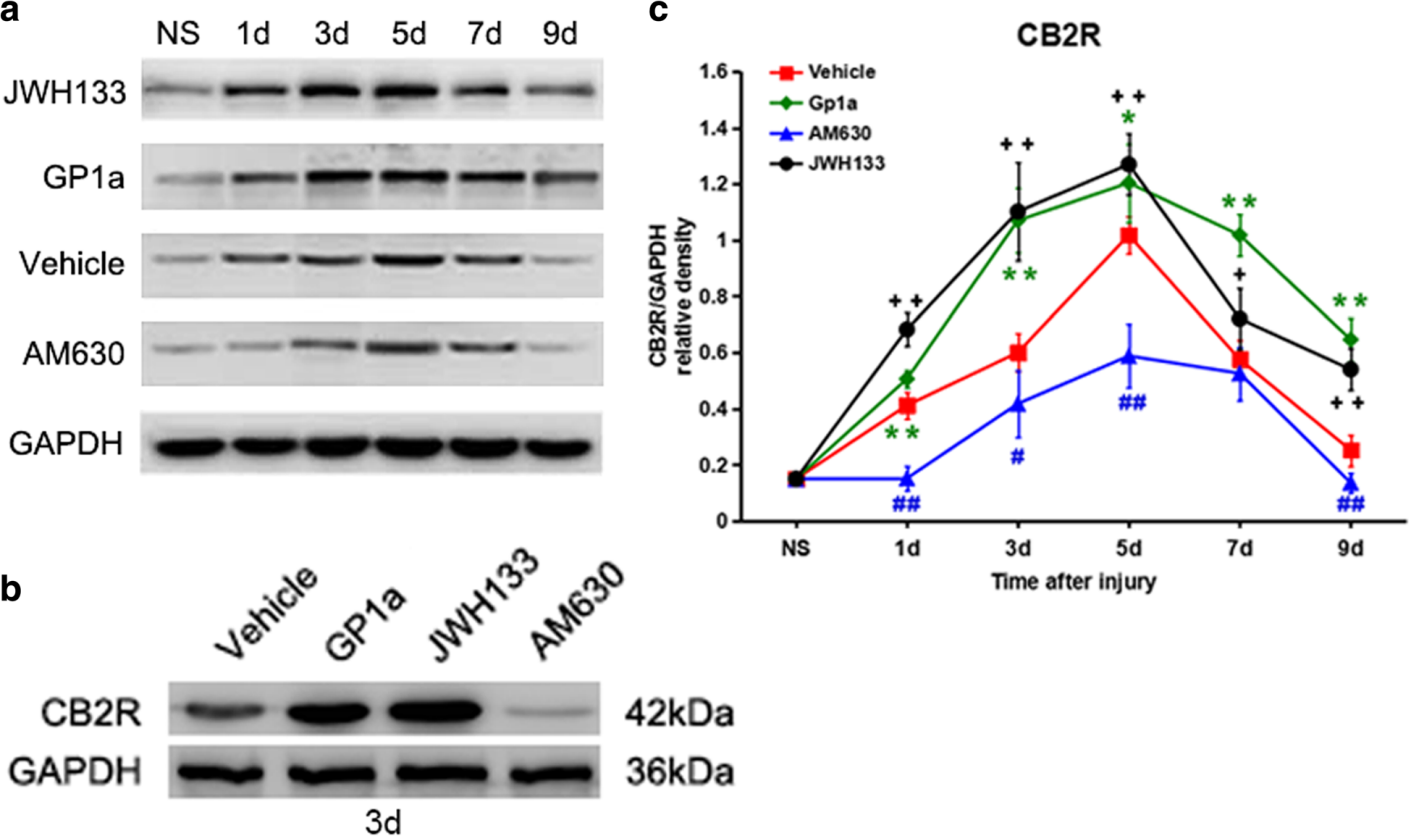
**a and b** Representative immunoblotting results and (**c**) semi-quantitative analysis of CB2R protein level at different posttraumatic intervals. ^+^ *P* < 0.05, ^++^ *P* < 0.01, JWH133 versus vehicle; * *P* < 0.05, ** *P* < 0.01, GP1a versus vehicle; ^#^ *P* < 0.05, ^##^ *P* < 0.01, AM630 versus vehicle

Blots of proteins from skin samples using GAPDH and CB2R antibodies are shown in Fig. 1a and b. For the vehicle control group, the CB2R protein level was up-regulated 1 day after injury (*P* < 0.01 vs. nonsurgical group), reached a peak after 5 days (*P* < 0.01 vs. nonsurgical group), and decreased from 7 to 9 days post-injury (*P* < 0.01 vs. nonsurgical group). After treatment of mice with JWH133 or GP1a, the protein level of CB2R increased significantly, with a statistical difference compared with the vehicle control group at each post-injury interval from 1 to 9 days (*P* < 0.05 vs. vehicle group). In contrast, administration of the antagonist caused the CB2R protein level to decrease substantially (*P* < 0.05 vs. vehicle group; Fig. 1c). These results indicate that the CB2R protein level increases in skin after injury.

### Effect of CB2R modulation on the infiltration of M1/M2 macrophages in skin lesions

**Fig. 2:**
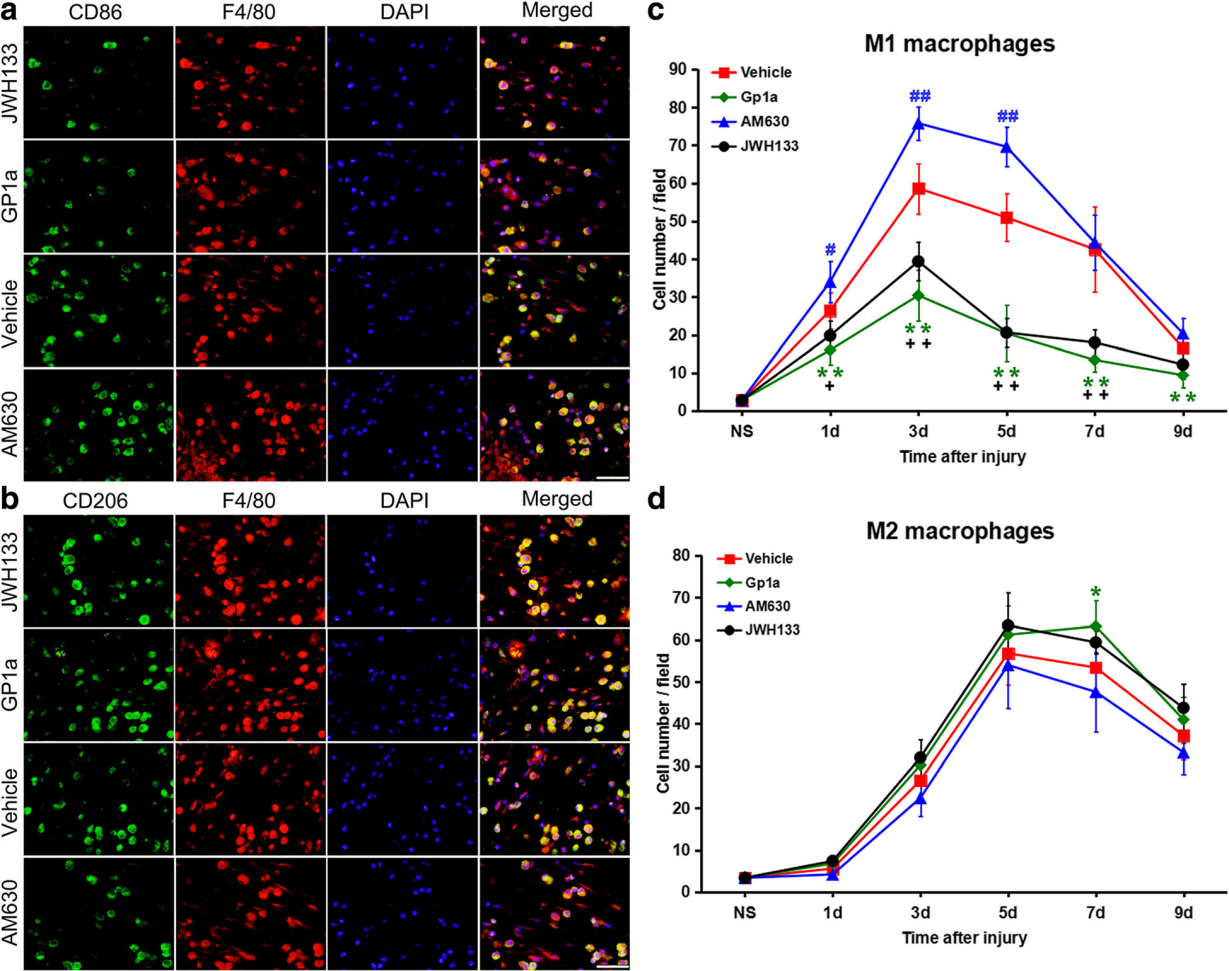
Macrophages infiltration alteration after JWH133, GP1a or AM630 treatment. **a and b** Representative immunofluorescence stains of M1 macrophages (CD86 green color; F4/80 red color; nuclei blue color) and M2 macrophages (CD206 green color; F4/80 red color; nuclei blue color) in the wound zone at 5 days post-injury. Scale bar = 50 μm. **c and d** Cell counting changes of M1 and M2 macrophage after JWH133, GP1a or AM630 treatment. ^+^ *P* < 0.05, ^++^ *P* < 0.01, JWH133 versus vehicle; * *P* < 0.05, ** *P* < 0.01, GP1a versus vehicle; ^#^ *P* < 0.05, ^##^ *P* < 0.01, AM630 versus vehicle

To examine whether the modulation of CB2R affected macrophage polarisation after injury, we first measured the number of M1 (F4/80^+^and CD86^+^) and M2 (F4/80^+^ and CD206^+^) macrophages infiltrating skin lesions by immunofluorescence staining (Fig. 2a and b).

In the vehicle group, the infiltration of M1 macrophages increased markedly 1 day post-injury (*P* < 0.01 vs. nonsurgical group), peaked at 3 days (*P* < 0.01 vs. nonsurgical group), and decreased over subsequent days. The number of M2 macrophages also increased rapidly at 3 days (*P* < 0.01 vs. nonsurgical group), became maximal at 5 days (*P* < 0.01 vs. nonsurgical group), then gradually decreased at 7 and 9 days (*P* < 0.01 vs. nonsurgical group). In a comparison, greater numbers of M1 macrophages than M2 macrophages were observed 1 and 3 days post-injury, while M2 macrophages replaced M1 macrophages as the predominant macrophage in the wound from 5 days post-injury.

Following JWH133 or GP1a treatment, the number of M1 macrophages was significantly reduced 1–9 days post-injury (*P* < 0.01 vs. vehicle group). In contrast, after treatment with AM630, the number of M1 macrophages increased significantly 1–5 days post-injury (*P* < 0.05 vs. vehicle group; Fig. 2c). As for M2 macrophages, after the administration of JWH133 or GP1a, a slight increase in the number 1–9 days post-injury was observed compared with those of the vehicle group, but a significant difference was only found at 7 days (*P* < 0.05 vs. vehicle group). For the AM630 group, the number of M2 macrophages slightly decreased 1–9 days post-injury; however, a significant difference was not observed compared with the vehicle group (Fig. 2d).

These results indicate that both M1 and M2 macrophages infiltrate an injury. However, M1 macrophages predominate early after an injury while M2 macrophages are predominant in the later stages. In addition, infiltration by M1 macrophages is likely in response to the expression of CB2R.

### Effect of CB2R modulation on gene expression of M1/M2-associated markers and cytokines

To examine whether CB2R affected macrophage polarisation and inhibited inflammation after a skin incision, we measured mRNA levels of M1 macrophage activated markers (CD86, iNOS) and secreted pro-inflammatory cytokines (IL-6, IL-12), as well as M2 macrophage activated markers (CD206, Arg-1) and anti-inflammatory secreted cytokines (IL-4, IL-10), respectively, using RT–qPCR.

**Fig. 3:**
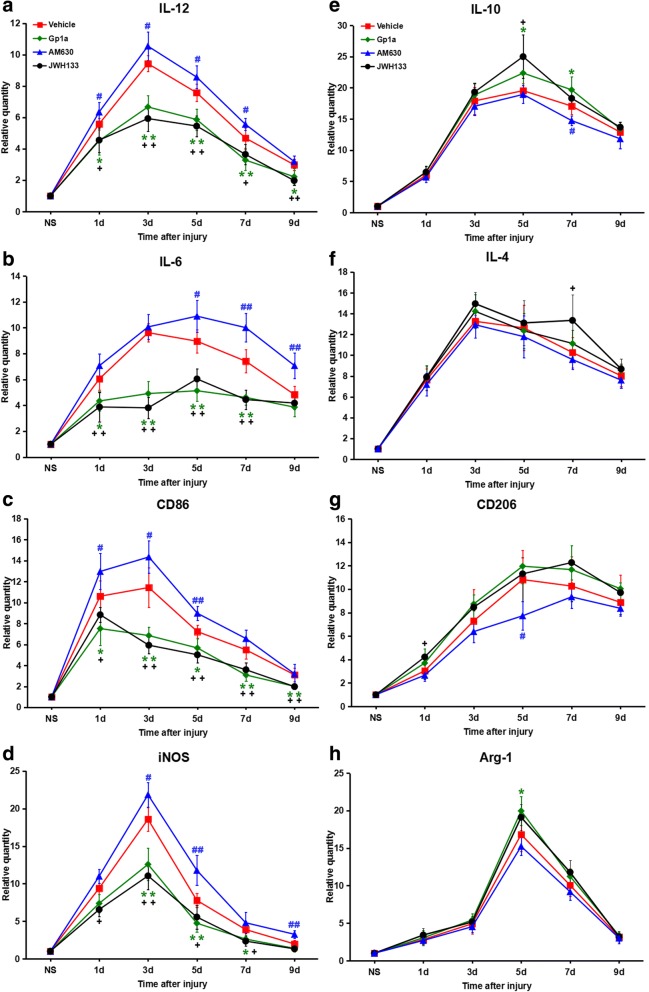
M1/M2-associated gene expression changes after JWH133, GP1a or AM630 treatment. **a-d** M1-associated mRNA expressions, including IL-6, IL-12, CD86 and iNOS. **e-h** M2-associated mRNA expressions, including IL-4, IL-10, CD206 and Arg-1. Data are mean ± SD. ^+^ *P* < 0.05, ^++^ *P* < 0.01, JWH133 versus vehicle; * *P* < 0.05, ** *P* < 0.01, GP1a versus vehicle; ^#^ *P* < 0.05, ^##^ *P* < 0.01, AM630 versus vehicle

For the vehicle group, the dynamics of M1-associated markers and cytokine mRNA levels was consistent with the number of M1 macrophages observed at wound edges. That is, the mRNA levels of IL-6, IL-12, CD86 and iNOS were up-regulated markedly 1 day post-injury, peaked at 3 days, then gradually decreased from 5 to 9 days post-injury. The mRNA levels of IL-12 were strikingly decreased in JWH133- or GP1a-treated mice, being statistically significant 1, 3, 5, 7, and 9 days post-injury (*P* < 0.05 vs. vehicle group). In contrast, the IL-12 level was significantly elevated in mice treated with AM630, being statistically significant 1, 3, 5, and 7 days post-injury (*P* < 0.05 vs. vehicle group; Fig. 3a). The dynamics of IL-6, CD86 and iNOS mRNA levels after the administration of an agonist or antagonist (Fig. 3b-d) were similar to those shown for IL-12.

We found that, compared with the vehicle group, the mRNA levels of M2-associated markers and cytokines (IL-4, IL-10, CD206, Arg-1) were increased slightly after JWH133 or GP1a administration, and decreased slightly by treatment with AM630, consistent with their dynamics during M2 macrophage infiltration. However, statistical differences were not found in the relative quantity of M2-associated markers and cytokines either agonist or antagonist and vehicle groups at most time points post-injury, as shown in Fig. 3e-h.

These results indicate that M1-associated marker and cytokine mRNA levels increased and then decreased after a skin injury, reflecting M1 macrophage infiltration seen over time.

### Effect of CB2R modulation on protein levels of M1/M2-associated markers and cytokines

Protein levels of M1/M2-associated markers and cytokines were detected in all skin samples, including controls, by ELISA assays. For each marker or cytokine, the dynamics of protein expression were consistent with those of the mRNA.

**Fig. 4:**
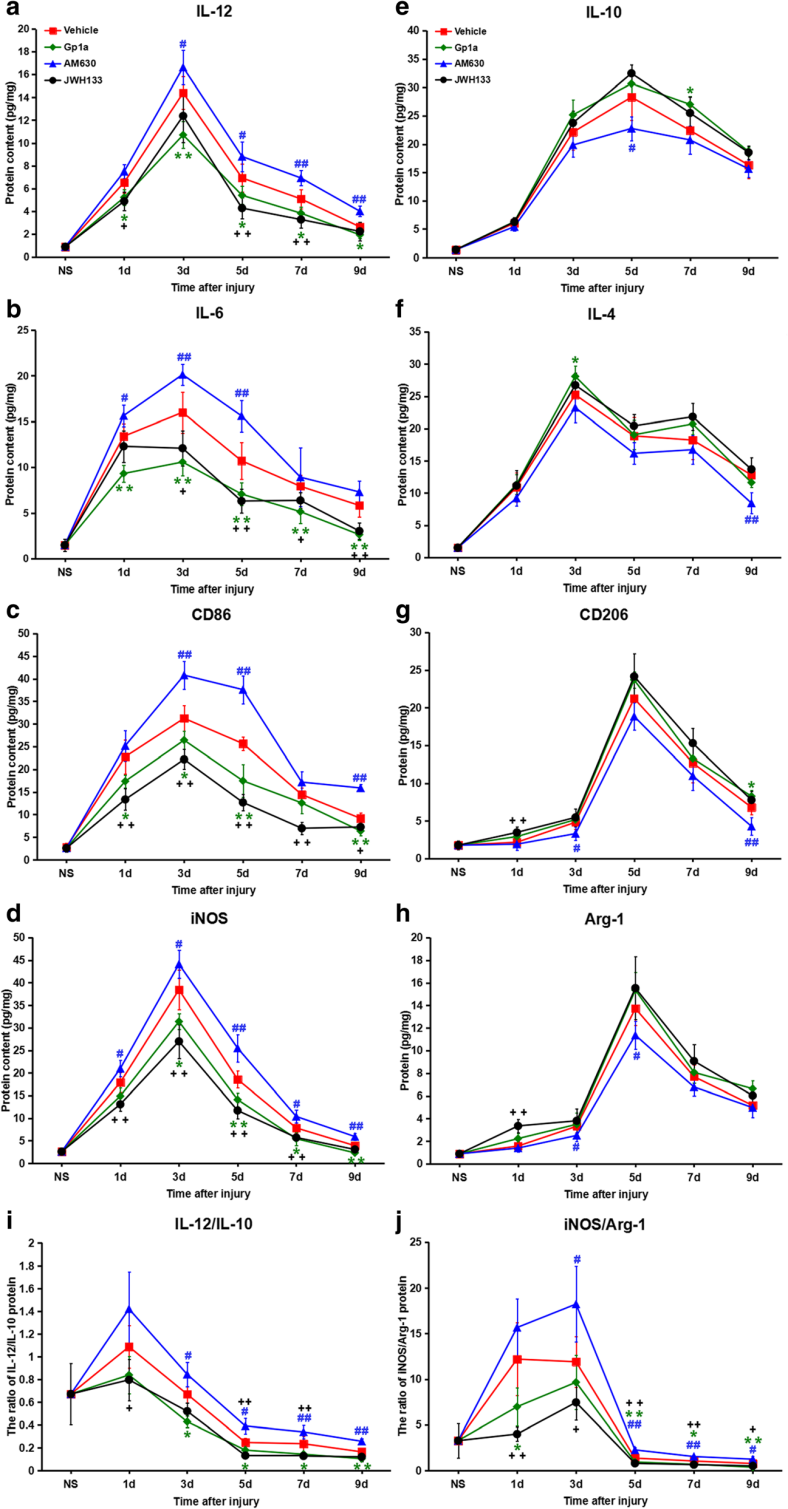
M1/M2-associated protein level changes after JWH133, GP1a or AM630 treatment. **a-d** M1-associated protein levels, including IL-6, IL-12, CD86 and iNOS. **e-h** M2-associated protein levels, including IL-4, IL-10, CD206 and Arg-1. **i and j** The iNOS/Arg-1 and IL-12/IL-10 protein level. Data are mean ± SD. ^+^ *P* < 0.05, ^++^ *P* < 0.01, JWH133 versus vehicle; * *P* < 0.05, ** *P* < 0.01, GP1a versus vehicle; ^#^ *P* < 0.05, ^##^ *P* < 0.01, AM630 versus vehicle

Protein levels of IL-6, IL-12, CD86 and iNOS were down-regulated significantly in the agonist group for which significant differences were found at most time points post-injury (*P* < 0.05 vs. vehicle group), whereas, in contrast, the antagonist group showed significant upregulation of these proteins at most time points post-injury (*P* < 0.05 vs. vehicle group; Fig. 4a–d). Compared with the vehicle group, the protein levels of IL-4, IL-10, CD206 and Arg-1 were increased in JWH133- or GP1a-treated mice and decreased in AM630-treated mice; however, significant differences at most time points post-injury were not noted (Fig. 4e–h).

We next evaluated the dynamic of M1/M2 in an approximate manner according to iNOS/Arg-1 and IL-12/IL-10 protein levels. The IL-12/IL-10 protein level was 0.67 in the control (nonsurgical) group; this then significantly increased to 1.09 (*P* < 0.05 vs. nonsurgical group) for the vehicle group 1 day post-injury. The ratio of proteins was almost back to the level of the control group 3 days after injury, and thereafter significantly decreased over subsequent days, to < 0.25 for all (*P* < 0.01 vs. nonsurgical group). Compared to that of the vehicle group, the IL-12/IL-10 protein level was significantly attenuated in agonist-treated groups, whereas this was elevated significantly in the antagonist-treated group. Significant differences were found 1, 3, 5, 7 and 9 days post-injury in the agonist-treated group (*P* < 0.05 vs. vehicle group), and at 3, 5, 7 and 9 days post-injury in the antagonist group (*P* < 0.05 vs. vehicle group) as shown in Fig. 4i.

The iNOS/Arg-1 protein level was 12.2 at 1 day (*P* < 0.01 vs. nonsurgical group) and 11.9 at 3 days (*P* < 0.01 vs. nonsurgical group) post-injury; the corresponding value in the control group was 2.58. The ratio was < 1.50 5, 7 and 9 days post-injury (*P* < 0.01 vs. nonsurgical group) and reached its lowest level at 9 days (0.76). Similarly, the administration of JWH133 or GP1a significantly attenuated the iNOS/Arg-1 protein level, whereas this ratio was significantly elevated in mice treated with AM630; significant differences were found at most time points post-injury (*P* < 0.05 vs. vehicle group; Fig. 4j).

These results indicate that M1-associated markers and cytokine protein levels were detected after a skin injury and that these were likely regulated by CB2R. Also, although both M1 and M2 macrophage markers and cytokines were detected after a skin injury, these indicated M1 macrophages were likely to be present in greater numbers post-injury.

## Discussion

The anti-inflammatory properties of CB2R in injury and inflammatory diseases have been widely studied and shown [5, 6, 8–11]. Our previous study demonstrated that during excisional wound healing in skin, activating CB2R markedly attenuated inflammation, whereas inhibiting CB2R enhanced the inflammatory reaction [8]. The results from this study further revealed that the anti-inflammatory effect of CB2R may be achieved by inhibiting pro-inflammatory M1 macrophage polarisation rather than activating anti-inflammatory M2 macrophages.

During skin wound healing, activated macrophages are primary participants in the inflammatory response [7, 8, 25] and key-regulators of wound repair [12]. More importantly, macrophages can regulate inflammation through their phenotypic polarisation according to different immune microenvironments [15–17]. In the early stages of inflammation, the M1 phenotype becomes activated through the classical pathway to promote inflammatory responses by releasing a series of pro-inflammatory cytokines and chemokines, such as IL-1β, IL-6, IL-12, IL-23, tumor necrosis factor-α, and iNOS [17, 26, 27]. However, persistent and excessive inflammatory responses can aggravate tissue injury, lead to chronic inflammation, and thereby prevent the routine process of tissue repair. To reduce inflammation and promote wound healing in the later stage of inflammation, macrophages are transformed into an M2 phenotype by an alternative activation pathway and secrete anti-inflammatory cytokines, such as Arg-1, IL-4, IL-10, transforming growth factor-β1, and Ym1 [17, 19, 26, 27]. Therefore, the dynamic equilibrium between M1 and M2 macrophages is vital for wound healing and tissue homeostasis.

In previous studies, the ratio of iNOS to Arg-1 mRNA levels has been used as an indicator of M1/M2 activity balance [28, 29]. In order to reflect the dynamics of M1/M2 after CB2R activation in skin wound healing, we measured iNOS/Arg-1 and IL-12/IL-10 protein levels in these studies. We found that after JWH133 or GP1a treatment, iNOS/Arg-1 and IL-12/IL-10 markedly decreased compared with those of the vehicle group. The implications of these results are as follows: (1) CB2R activation may have decreased the ratio of pro-inflammatory to anti-inflammatory cytokines around the wound, which alleviated the inflammation and was beneficial to wound healing; (2) Activated CB2R reduced the proportion of M1 subtypes in macrophages or increased the relative proportion of M2 subtypes; however, changes in the number of M1 or M2 macrophages cannot be accurately demonstrated.

To demonstrate the dynamics of infiltrating M1 or M2 macrophages, immunofluorescence staining was undertaken. We found that both M1 and M2 macrophages exhibited sequential quantitative changes during the healing of an incised wound in mouse skin. The number of M1 macrophages reached a peak 3 days post-injury, while M2 macrophages peaked after 5 days, which was consistent with the function of the different macrophage subtypes. That is, M1 macrophages play a pro-inflammatory role in the early stages of inflammation, and M2 macrophages promote tissue repair in the late stages of inflammation. Furthermore, M1 macrophages significantly decreased after CB2R activation in response to treatment with JWH133 or GP1a, whereas they increased significantly in the AM630-treated group, indicating that CB2R activation could significantly reduce M1 macrophage numbers around a skin incision. As an anti-inflammatory macrophage subtype, M2 macrophages were up-regulated by JWH133 or GP1a and down-regulated by AM630. However, compared with the vehicle group, neither the agonist nor antagonist group showed any statistical significance at most post-injury intervals, which suggested that the effect of CB2R modulation on the polarisation of M2 macrophages is not as significant as that on M1 macrophages. We previously showed that activated CB2R attenuated inflammation by reducing the infiltration of macrophages around the wound [8]. According to the results of the present study, we further propose that during incised skin wound healing in mice, reduced inflammation after CB2R activation was mainly achieved by inhibiting M1 polarisation, rather than by promoting M2 polarisation.

In order to further reveal the role of CB2R in regulating macrophage polarisation during skin wound healing, we measured gene expression and protein levels of M1/M2-associated markers and cytokines. In accordance with previous studies [19, 27, 30], we chose IL-6, IL-12, CD86 and iNOS as markers of M1 macrophage activation, and IL-4, IL-10, CD206 and Arg-1 as indicators of M2 macrophage responses. We showed that the expression of M1 macrophage markers (CD86, iNOS) and pro-inflammatory cytokines (IL-6, IL-12) was significantly decreased following treatment with JWH133 or GP1a, which indicated CB2R decreased the polarisation of macrophages to the M1 phenotype and inhibited the release of pro-inflammatory cytokines, thus reducing inflammation during skin wound healing. Meanwhile, activation or inhibition of CB2R may lead to a corresponding increase or decrease, respectively, in the expression of M2 phenotype markers (CD206, Arg-1) and anti-inflammatory cytokines (IL-4, IL-10). However, significant differences at most post-injury intervals were not observed, which suggested that the effect of CB2R on M2 macrophage polarisation at gene and protein levels was not significant after injury.

To demonstrate the effect of activation or inhibition of CB2R on macrophage polarisation, we chose JWH133 [18, 27] and GP1a [8, 22, 31, 32] as CB2R agonists and AM630 [19, 27] as a CB2R antagonist based on previous reports and our studies. Importantly, the up- and down-regulation of CB2R and the alteration of M1/M2-related protein levels and gene expression after the administration of agonists or an antagonist were consistent with our expectations. We therefore have reason to believe that GP1a has an activating effect on CB2R. This is despite the controversy surrounding GP1a is an agonist of CB2R [33]. However, we consider that differences found by others with regard to the effect of GP1a on CB2R may have been related to differences in animal species or the tissues of experimental animals used.

The present results differ from those of others, which proposed that CB2R could significantly promote M2 macrophage polarisation after injury and then attenuate neuroinflammation [18–21]. Whether such a discrepancy in research results is due to the use of different animal models needs to be further examined. M2 macrophages promoting the development of fibrosis [17, 24] and CB2R reducing fibrosis [8, 22, 23] have been observed by others. Therefore, the relationship between of CB2R and M2 macrophages in fibrosis and tissue remodeling post-injury may be complicated and requires further study. For example, it would be useful to study the effect of CB2R on macrophage polarisation using CB2R knockout mice and to detect local endogenous cannabinoid levels, subjects that will be the foci of future studies.

## Conclusion

Taken together, our findings suggest that during incised skin wound healing in mice, the expression of CB2R may affect inflammation by regulating M1 rather than M2 macrophage subtype polarisation. The activation of CB2R and the promotion of M2 polarisation observed in injury offer different mechanisms by which inflammation may be inhibited; any combined therapy based on these two mechanisms may have an enhanced therapeutic effect on skin inflammation. These studies further our understanding of the molecular mechanisms involved in the CB2R inhibition of inflammation and may lead to novel treatments for cutaneous inflammation.
